# Anemia as a risk factor for tuberculosis: a systematic review and meta-analysis

**DOI:** 10.1186/s12199-020-00931-z

**Published:** 2021-01-23

**Authors:** Yemataw Gelaw, Zegeye Getaneh, Mulugeta Melku

**Affiliations:** Department of Hematology and Immunohematology, College of Medicine and Health Sciences, School Biomedical and Laboratory Sciences, University of Gondar, Gondar, Ethiopia

**Keywords:** Anemia, Tuberculosis, Systematic review, Hazard ratio, and Meta-analysis

## Abstract

**Background:**

Tuberculosis is a major public health problem caused by *Mycobacterium tuberculosis*, occurring predominantly in population with low socioeconomic status. It is the second most common cause of death from infectious diseases. Tuberculosis becomes a double burden among anemic patients. Anemia increases an individual’s susceptibility to infectious diseases including tuberculosis by reducing the immunity level. Therefore, the purpose of this study was to determine whether anemia is a risk factor for tuberculosis.

**Method:**

Relevant published articles were searched in electronic databases like PubMed, Google Scholar, EMBASE, and Cochrane Library using the following MeSH terms: risk factor, predictors, tuberculosis, TB, Anaemia, Anemia, hemoglobin, Hgb, and Hb. Articles written in the English, observational studies conducted on the incidence/prevalence of tuberculosis among anemic patients, or papers examined anemia as risk factors for tuberculosis were included. From those studies meeting eligibility criteria, the first author’s name, publication year, study area, sample size and age of participants, study design, and effect measure of anemia for tuberculosis were extracted. The data were entered using Microsoft Excel and exported to Stata version 11 for analysis. The random-effects model was applied to estimate the pooled OR and HR, and 95% CI. The sources of heterogeneity were tested by Cochrane I-squared statistics. The publication bias was assessed using Egger’s test statistics.

**Results:**

A total of 17 articles with a 215,294 study participants were included in the analysis. The odd of tuberculosis among anemic patients was 3.56 (95% CI 2.53–5.01) times higher than non-anemic patients. The cohort studies showed that the HR of tuberculosis was 2.01 (95% CI 1.70–2.37) times higher among anemic patients than non-anemic patients. The hazard of tuberculosis also increased with anemia severity (HR 1.37 (95% CI 0.92–2.05), 2.08 (95% CI 1.14–3.79), and 2.66 (95% CI 1.71–4.13) for mild, moderate, and severe anemia, respectively).

**Conclusion:**

According to the current systematic review and meta-analysis, we can conclude that anemia was a risk factor for tuberculosis. Therefore, anemia screening, early diagnose, and treatment should be provoked in the community to reduce the burden of tuberculosis.

## Background

Anemia, defined as low blood hemoglobin (Hb) concentration (less than 11.0 g/dl for 6–59-month children, 11.5 g/dl for 5–11-year-old children, 12.0 g/dl for 12–14-year-old children and non-pregnant women (for age 15 years and above), 11.0 g/dl for pregnant women, and 13.0 g/dl for adult men (for age 15 years and above). It is a global public health problem affecting both developing and developed countries. It occurs at all stages of the life cycle, but is more prevalent in pregnant women and young children [1, 2]. Globally, it affects 24.8% of the population [3] with the highest prevalence occurred in preschool-age children (43%) [1]. It is the result of a wide variety of causes in which 50% of the cases are due to iron deficiency. Acute and chronic infections, including malaria, cancer, and HIV are also the cause of anemia [1, 3].

Anemia is classified into three categories based on the severity; mild, moderate, and severe anemia. Mild anemia is defined as Hb concentration of 10.0–10.9 g/dl for pregnant women and 6–59-month children, 11.0–11.4 g/dl for 5–11-year-old children, 11.0–11.9 g/dl for non-pregnant women, 12–14-year-old children, and 11.0–12.9 g/dl for adult men. On the other hand, moderate anemia defined as the Hb value of 7.0–9.9 g/dl for pregnant women and 6–59-month children and 8.0–10.9 g/dl for 5–11-year-old children; 12–14-year-old children, non-pregnant women, and adult men while severe anemia is defined as the Hb value less than 7.0 g/dl for pregnant women and 6–59 months of children; and less than 8.0 g/dl for 5–11-year-old children, 12–14-year-old children, non-pregnant women, and adult men [2].

Anemia reduces health-related quality of life, increases morbidity and mortality in patients with chronic disease. It also predisposes an individual to some infectious diseases including tuberculosis (TB) [4]. It is a common hematological finding among TB patients with the prevalence of 44–89.1% [5–9]. On the other hand, the proportion of TB among anemic patients is higher than non-anemic patients (the highest burden in severe anemic patients) [10, 11]. In anemic patients, cell-mediated immune response and bactericidal capacity of leucocytes are significantly suppressed [12, 13].

Tuberculosis is an airborne chronic infectious disease caused by *Mycobacterium tuberculosis* and predominantly occurred in low socio-economical segments of population. It is the second most common cause of death among infectious diseases. A total of 8.7 million new active TB cases and 1.4 million TB-related deaths were estimated globally [14–16].

The assessment of potentially modifiable risk factors is a vital for the development of TB control policies [17]. Accordingly, some TB risk factors have been known for decades, including systemic diseases such as diabetes mellitus and chronic kidney disease as well as tobacco smoking, alcohol use, body mass index, silicosis, human immunodeficiency virus (HIV) infection, splenectomy, and gastrectomy [17, 18]. Under-nutrition, refugee, homeless, and direct contact with active TB are also risk factors for TB [18]. People exposed to these factors are called the risk groups for TB in which the prevalence or incidence of TB is significantly higher than in the general population. The World Health Organization (WHO) recommended and established guidelines for these risk groups to be prioritized for screening of active TB than the general population [19].

However, even the diagnosis of anemia with Hb measurement is a low cost, and more widely available in clinical settings to know the anemic status of the individual [20], there is no any established guideline or policies to consider anemic patients as the risk group for TB and to be prioritized them for screening for active TB. But, studies have investigated the link between anemia and TB prevalence (the relationship between anemia and the risk of contracting TB). Accordingly, some studies showed that anemia is risk for TB [21–23]. In contrast, others showed that anemia is not the risk of development of TB [20, 24]. These contradict findings and the absence of systemic review and meta-analysis conducted about the risk of contracting TB among anemic patients; motivate the authors to conduct this systemic review and meta-analysis. Thus, the main objective of the current systematic review and meta-analysis was to determine the pooled risk effect of anemia on the development TB.

## Methods

### Design and protocol registration

This systematic review and meta-analysis was designed according to the Preferred Reporting Items for Systematic Reviews and Meta-Analysis Protocols (PRISMA-P 2015 Guidelines) [25]. The protocol has been registered in the PROSPERO with the registration number of CRD42019161729.

### Inclusion and exclusion criteria

Articles wrote only in the English language; observational studies like cohort studies, case-control studies, and cross-sectional studies; conducted on the incidence/prevalence of TB among anemic patients (any type of anemia) or papers assessed anemia as risk factors for TB, human studies, and articles published until December 17, 2019; and available on the searched databases were included. We excluded studies that did not report crude or adjusted HR, crude or adjusted RR, crude or adjusted OR, or unable to calculate these effect measures from 2 by 2 tables.

### Searching strategy and research screening

The articles for this systematic review and meta-analysis were retrieved through reproducible and comprehensive electronic searching of major reputable databases (PubMed, EMBASE, and Cochrane Library) and Web search (Google Scholar and Science direct) using the following MeSH terms: tuberculosis, TB, Anaemia, Anemia, Hemoglobin, and Hgb, Hb, risk factor, and predictors. We also did a manual searching of reference lists of already identified relevant articles to retrieve more eligible studies by using the combination of these keywords. The two authors (YG, MM) performed the search independently based on the following key terms: [1] population (anemia, anaemia, hemoglobin, Hgb Hb), [2] outcome (tuberculosis, TB), [3] study design (prevalence, incidence, cross-sectional, observational, cohort study), and [4] location (worldwide). The terms were used both separately and in combination with the help of the Boolean operator like “AND”, “OR”. We looked for these terms in the abstract, title, or keywords. Finally, the search results were imported to an endnote to find duplicates. Titles and abstracts were examined by 3 independent reviewers (YG, ZG, and MM). Besides, articles referenced by those identifiers were reviewed for relevance.

### Study selection and quality appraisal

The quality appraisal of full-length original research articles was assessed in detail by two independent reviewers (ZG and MM) by using the Joanna Brigg Institute (JBI) criteria [26]. The JBI checklist items related to design, setting, participants, and confounders, bias, statistical analysis, outcome measures, results, and generalizability of the study were checked. The independent reviewers sat together and settled the differences in rating through consensus, before the final decision to include or exclude the articles. The scoring system is as follows: 0 (not done), 1 (done), UC (unclear), and NA (not applicable). The score range for this tool is between 0 (lowest quality) to 8 (highest quality) for cross-sectional, 0 (lowest quality) to 10 (highest quality) for case-control studies, and 0 (lowest quality) to 11 (highest quality) cohort studies. Articles with average score of 50% and above were included into this study.

### Data extraction

For each study, meeting eligibility criteria, the first author’s name, publication year, study area/country, sample size and age of participants as adult and children, study design, and results like crude or adjust HR or crude or adjust OR were extracted by YG using Microsoft Excel. The anemia severity status was also extracted according to the individual study report (Table 1). The logarithm of HR (log HR) and standard error (Se log HR), or logarithm of OR (log OR), and standard error (Se log OR) were calculated from their corresponding effect measure.

**Table 1:** Summary of the studies used in the systematic review and meta-analysis

Author/year	Study design	Country	Sample size	OR/HR (95% CI)	Source of population	Age of participant	Anemia severity
Lienhardt et al./2005# [27]	Case-control	West Africa	1,166	3.50* (2.57–4.79)	Community	Adult	Mild
Lienhardt et al./2005# [27]	Case-control	West Africa		10.80* (6.80–17.20)	Community	Adult	Moderate and severe
Taha et al./2011# [28]	Case-control	Ethiopia	809	1.87 (1.576–2.17)	HIV +	Adult	Mild
Taha et al./2011# [28]	Case-control	Ethiopia		4.35 (3.97–4.72)	HIV +	Adult	Moderate and severe
Kerkhoff et al./2014## [11]	Cross-sectional	S.Africa	485	2.05* (1.48–2.62)	HIV +	Adult	Mild
Kerkhoff et al./2014## [11]	Cross-sectional	S.Africa		3.65* (3.16–4.12)	HIV +	Adult	Moderate
Kerkhoff et al./2014## [11]	Cross-sectional	S.Africa		6.91* (6.05–7.78)	HIV +	Adult	Severe
Pefura et al./2013 [29]	Cross-sectional	Cameroon	857	1.60 (1.03–2.50)	PTB +	Adult	Not defined
Iroezindu et al./2016 [24]	Cross-sectional	Nigeria	339	4.50 (0.60–31.70)	HIV +	Adult	Not defined
Beshir et al./2019 [10]	Cohort	Ethiopia	428	7.04 (1.03–48.15)	HIV +	Children	Not defined
Ayalaw et al./2015 [30]	Cohort	Ethiopia	271	2.23 (1.19–4.15)	HIV +	Children	Not defined
Batista et al./2013[31]	Cohort	Brazil	1,596	2.93 (1.86–4.62)	HIV +	Adult	Not defined
Chu et al./2019 [17]	Cohort	Taiwan	109,501	1.99 (1.77–2.25)	Community	Adult	Not defined
Alemu et al./2016 [22]	Cohort	Ethiopia	645	2.70 (1.60–4.50)	HIV +	Children	Not defined
McDermid et al./2013 [32]	Cohort	Gambia	1,139	1.14 ** (1.02–1.27)	HIV +	Adult	Not defined
Li et al./2013 [33]	Cohort	Tanzania	5,040	1.40** (1.00–1.90)	HIV +	Children	Not defined
Enju et al./2015## [21]	Cohort	Tanzania	67,686	1.22 (1.13–1.33)	HIV +	Adult	Mild
Enju et al./2015## [21]	Cohort	Tanzania		1.66 (1.55–1.79)	HIV +	Adult	Moderate
Enju et al./2015## [21]	Cohort	Tanzania		2.03 (1.86–2.22)	HIV +	Adult	Severe
Kerkhoff et al./2015## [20]	Cohort	S.Africa	1,521	0.96*(0.72–1.63)	HIV+	Adult	Mild
Kerkhoff et al./2015## [20]	Cohort	S.Africa		1.27*(0.99–1.62)	HIV+	Adult	Moderate
Kerkhoff et al./2015## [20]	Cohort	S.Africa		1.22*(0.94–1.58)	HIV+	Adult	Severe
Phyo et al./2019## [23]	Cohort	Myanmar	7,859	2.10 (1.70–2.80)	HIV+	Adult	Mild
Phyo et al./2019## [23]	Cohort	Myanmar		4.30 (3.40–5.40)	HIV+	Adult	Moderate
Phyo et al./2019## [23]	Cohort	Myanmar		4.90 (3.30–7.20)	HIV+	Adult	Severe
Chang et al./2015## [34]	Cohort	Nigeria	12,996	2.48* (2.02–3.03)	HIV+	Adult	Mild and moderate
Chang et al./2015## [34]	Cohort	Nigeria		4.36*(1.98–9.62)	HIV+	Adult	Severe
Choun et al./2013## [35]	Cohort	Cambodia	2,956	2.10 (1.40–3.30)	HIV+	Adult	Mild and moderate
Choun et al./2013## [35]	Cohort	Cambodia		3.40 (2.10–5.50)	HIV+	Adult	Severe

*NB* * crude odds ratio for cross-sectional and case-control studies, and crude hazard ratio for cohort studies, **adjusted risk ratio and those without * or ** are adjusted odd ratio for cross-sectional and case-control studies, and adjusted hazard ratio for cohort studies, **#** the data split into two, ## the data split into three; *HIV+* human immunodeficiency virus positive patients; *HR* hazard ratio; *RR* relative risk; *OR* odds ratio; *PTB* pulmonary—tuberculosis patient; *S. Africa* South Africa

### Data analysis and interpretation

The extracted data were entered into Microsoft Excel and export to Stata version 11 for analyzing. The data analysis was by two authors (YG and MM). The potential source of heterogeneity across studies was tested by Cochrane *I*^2^ test statistics which shows the amount of heterogeneity between studies. The *I*^2^ provides the percentage of variability due to heterogeneity rather than chance difference or sampling error. *I*^2^ > 50% was considered statistically significant heterogeneity. The random-effects model which assesses the variability within and between studies was applied to estimate the pooled OR and HR and 95% confidence intervals (CIs). The publication bias was assessed using Egger’s test statistics with *p* value < 0.05 considered as the presence of publication bias. A sensitivity test was done to give a quick indication which study is the prime determinant of the pooled effect size and which is the main source of heterogeneity. The test excludes each study one by one in the analysis to show the pooled effect sizes and associated heterogeneity. Subgroup analysis was done by study design for cross-sectional and case-control studies.

## Results

### Description of studies

**Fig. 1:**
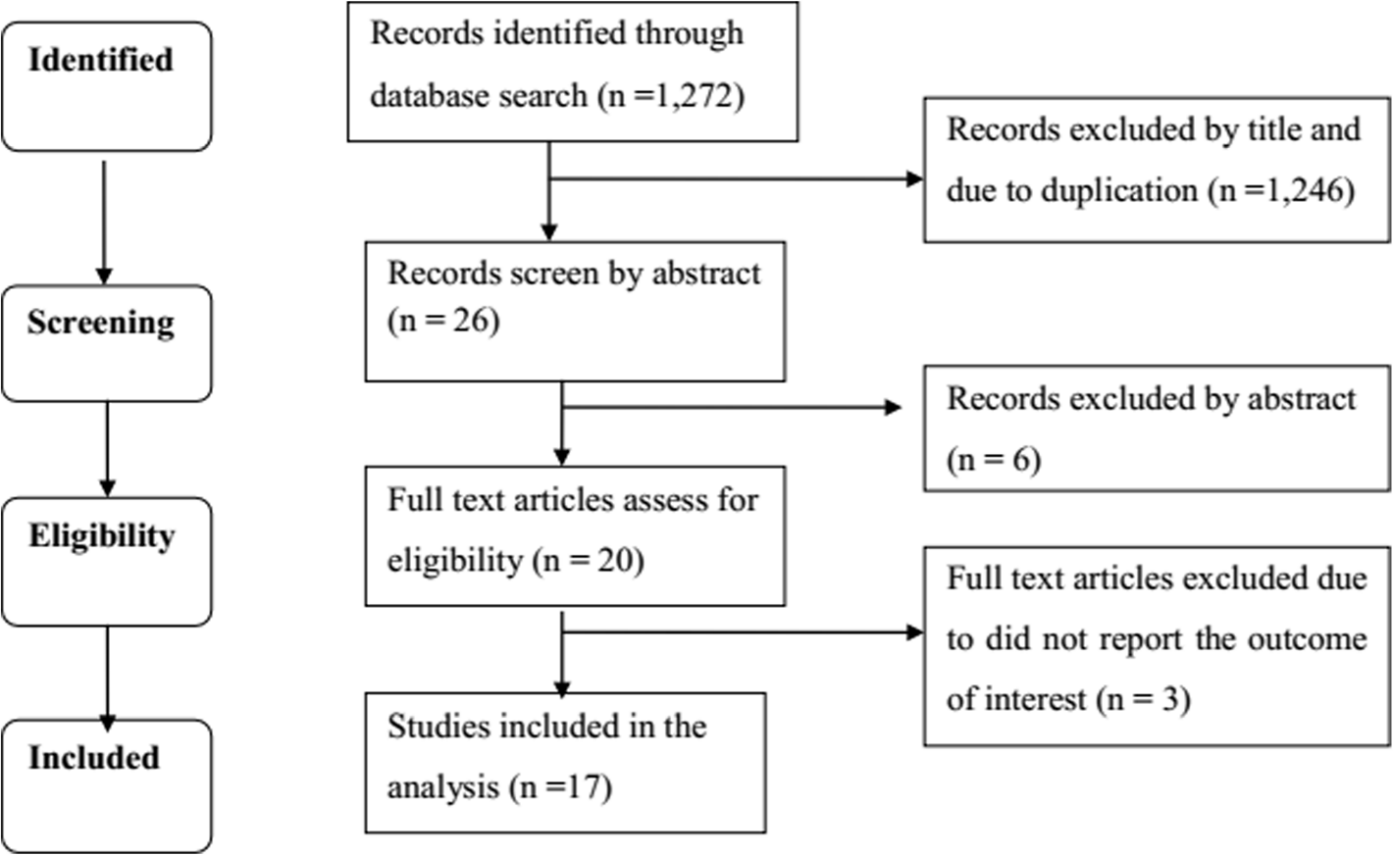
PRISMA flow chart of study selection. PRISMA: Preferred Reporting Items for Systematic Reviews and Meta-Analysis

Our initial search yielded 1272 articles. After 13 duplicates were removed, 1233 articles were excluded because of not relevant title, and 6 articles were excluded after reading the abstract. Finally, 20 articles identified for further assessment. Two studies that did not report the effect measures (HR, RR, OR) or unable to calculate these measures [36, 37], and one study conducted on latent TB [38] were also excluded. Finally, 17 studies were used for Meta-analysis (Fig. 1). Eight of the research articles included for analysis contained information on the association of anemia severity and TB development (5 cohort studies, 2 case-control studies, and 1 cross-sectional study). As a result, these were treated as two or three separate studies considering the anemia severity as a separate independent variable.

Of the included 17 articles, 2 (11.8%) research articles were published in years of 2005 [27] and 2011 [28] (one in each year’s) while 5 (29.4%) of them were published in the year of 2013 [29, 31, 32, 33, 35] and 1 (5.9%) of the research articles was published in the year of 2014 [11]. Nine (52.9%) of the included articles were published between 2015 and 2019 (4 (23.5%) in 2015 [20, 21, 30, 34], 2 (11.8%) in 2016 [22, 24], and 3 (17.6%) in 2019 [10, 17, 23]). Three of the included studies were cross-sectional studies and were conducted in South Africa [11], Cameroon [29], and Nigeria [24]. The other 2 studies were case-control studies conducted in three West Africa countries [27] and Ethiopia [28]. The remaining 12 studies were cohort studies of which 8 (66.67%) were conducted in Africa (1 in south Africa [20], 2 in Tanzania [21, 33], 1 Nigeria [34], 1 Gambia [32], and 3 in Ethiopia [10, 22, 30]). The other 4 cohort studies were conducted in Taiwan [17], Brazil [31], Cambodia [35], and Myanmar [23]. Based on the study participant, all of the cross-sectional and case-control studies were conducted on adults. On the other hand, among the cohort studies, 4 studies were conducted on children and 8 were conducted on adults (Table 1). Concerning the quality appraisal of the included studies, most of the research articles was scored greater than 80%.

A total of 215,294 participants were included in the 17 included studies (1681 participants in cross-sectional studies, 1975 participant in case-control studies, and 211,638 participants in cohort studies).

### Publication bias

**Fig. 2:**
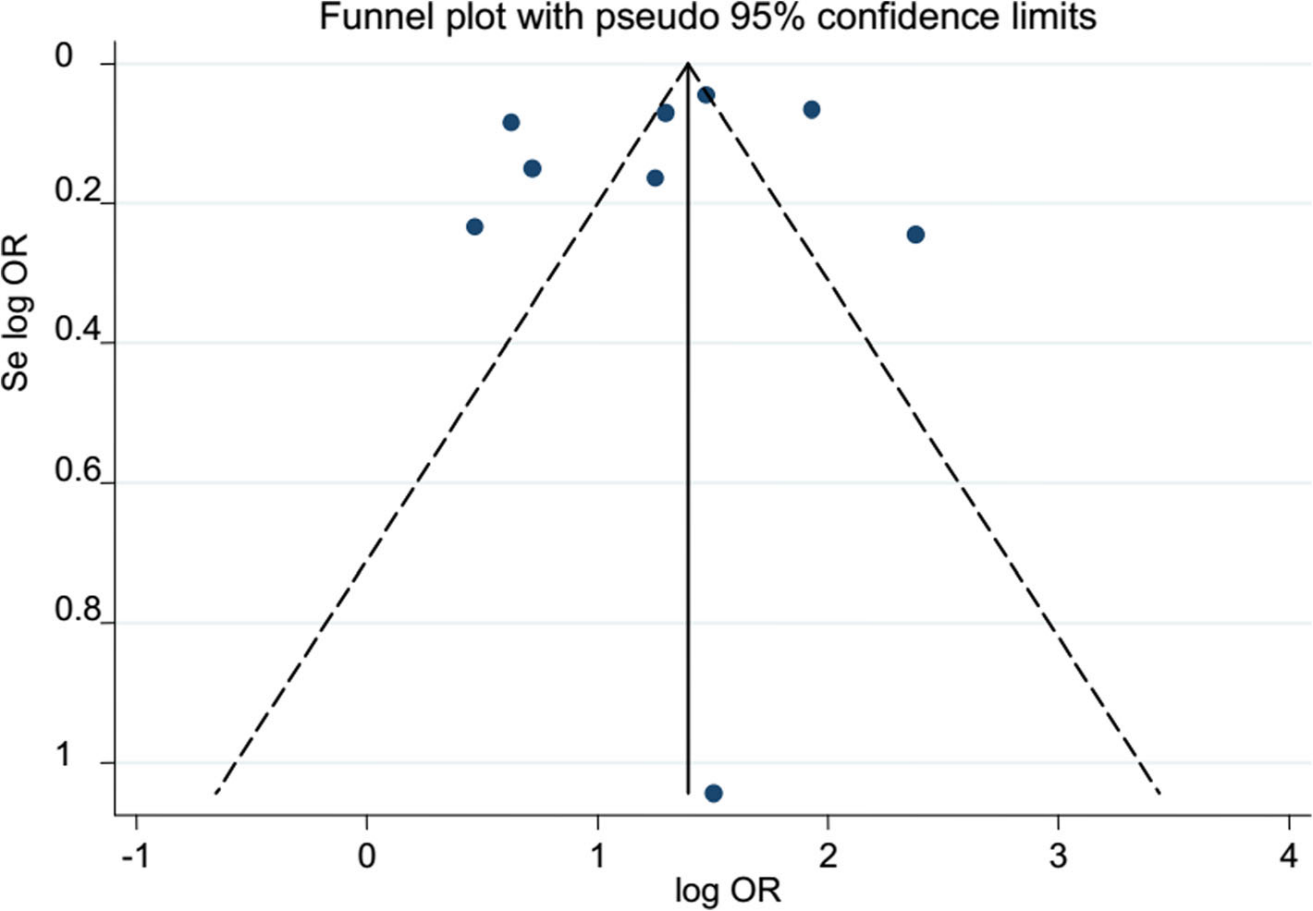
Funnel plot of case-control and cross-sectional studies; each dot represents individual studies. The *y*-axis represents standard error of estimate. The *x*-axis represents logit transformed estimates. log OR logarithm of odds ratio, Se log OR standard error of logarithm of odds ratio

**Fig. 3:**
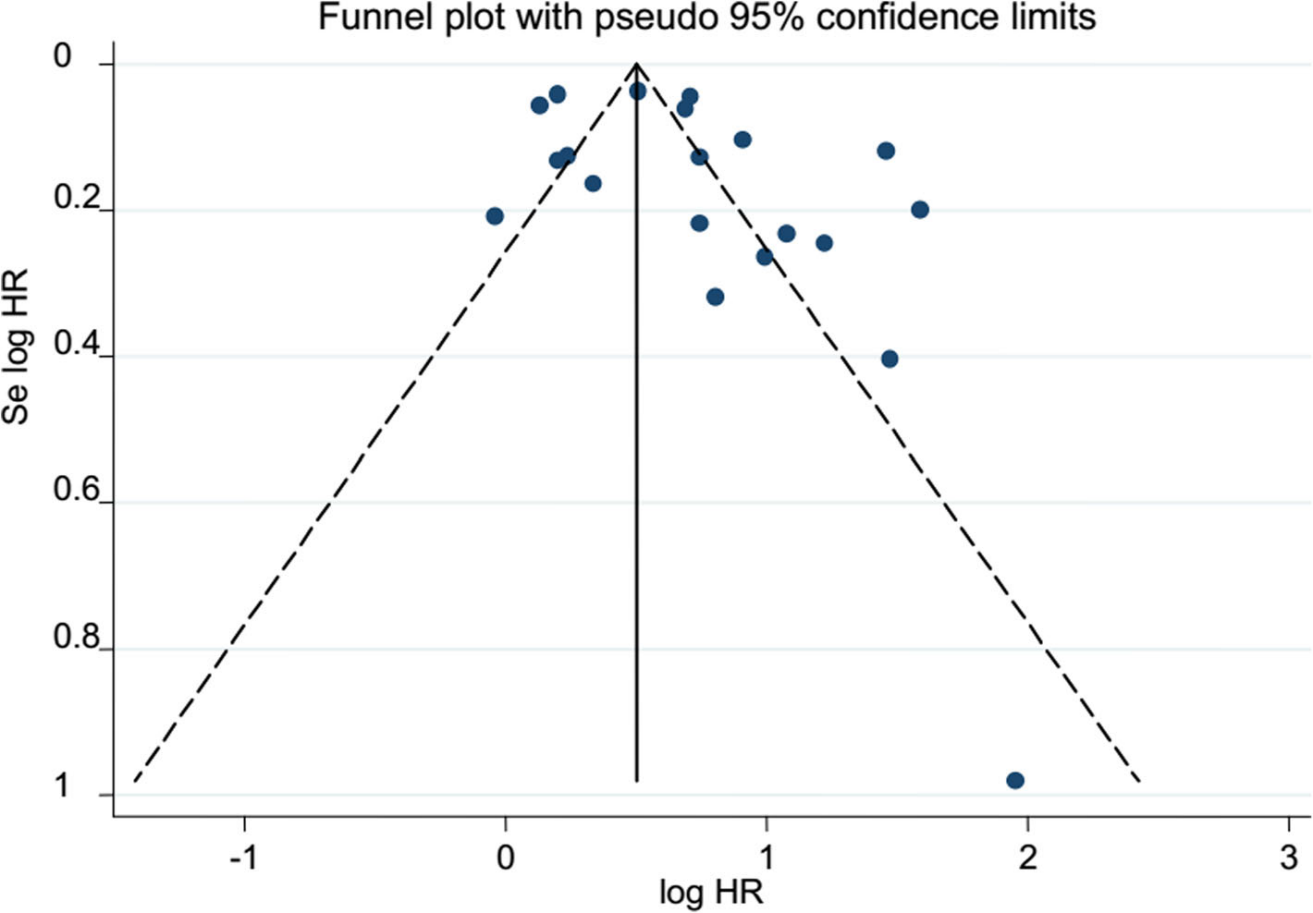
Funnel plot of cohort studies; each dot represents individual studies. The *y*-axis represents standard error of estimate. The *x*-axis represents logit transformed estimates. log HR logarithm of hazard ratio, Se log HR standard error of logarithm of hazard ratio

The included studies were assessed for publication bias based on their pooled analysis. The subjective assessment funnel plot of publication bias for case-control and cross-sectional studies (pooled by OR) and cohort studies (pooled by HR) looks asymmetrical which is an indication of publication bias (Figs. 2 and 3). However, Egger’s statistics test showed that there were no publication bias (Tables 2 and 3).

**Table 2:** Egger’s test for case-control and cross-sectional studies reported by OR

Std_Eff	Coef.	Std. Err.	*t*	*p* > |t|	(95% Conf. Interval)
Slope	1.54	0.276	5.57	0.001	(0.887–2.194)
Bias	− 2.028	3.145	− 0.64	0.540	(− 9.469–5.414)

*OR* odds ratio, *Std_Eff* standard effect, *Coef* coefficient**,** *Std.Err* standard error*, Conf. Interval* confidence interval, *t* stands for *t* test value, *p* indicates the statistical significance of the coefficient value assuming that null value is zero

**Table 3:** Egger’s test for cohort studies reported by HR

Std_Eff	Coef.	Std. Err.	*t*	*p* > |*t*|	(95% Conf. Interval)
Slope	0.352	0.109	3.22	0.005	(0.1224–0.5817)
Bias	2.335	1.318	1.77	0.093	(− 0.4347–5.105)

*HR* hazard ratio, *Std_Eff* standard effect, *Coef* coefficient, *Std.Err* standard error, *Conf. Interval* confidence interval, *t* stands for *t* test value, *p* indicates the statistical significance of the coefficient value assuming that null value is zero

### Sensitivity and heterogeneity test

**Fig. 4:**
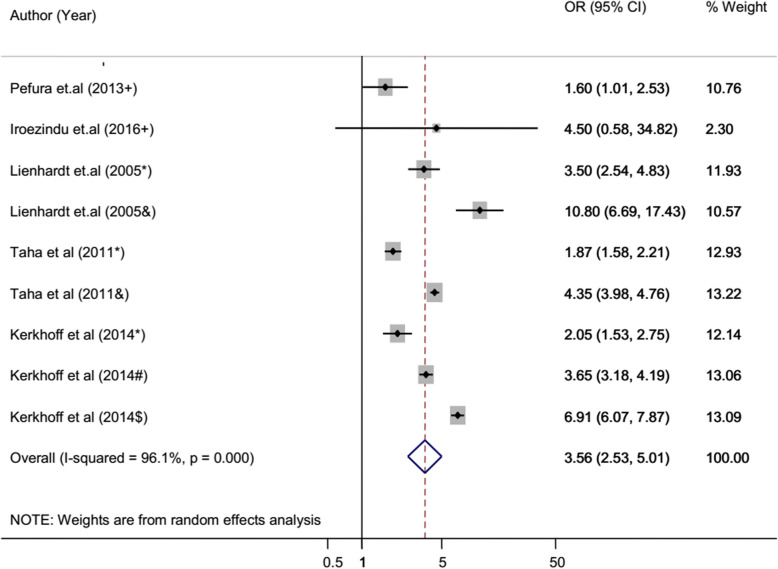
Forest plot of case-control and cross-sectional studies; pooled effect size (OR) estimates of anemia for the development of TB. The scale in the *x*-axis represents OR risk estimate of the studies. The hard line represents the OR value showing no association (OR of 1). The dashed line represents the pooled point estimate of OR of anemia for the TB infection. The black dot at the center of the gray box represents the point OR estimate of each study and the line indicates the 95% confidence interval of the estimates. The gray boxes represent the weight of each study contributing to the pooled OR estimate. The blue diamond represents the 95% confidence interval of the pooled OR estimates. OR odds ratio, CI confidence interval, I-squared shows the heterogeneity of the included studies, *p* indicates the statistical significance of the heterogeneity, + anemia severity not defined, * mild anemia, & moderate and severe anemia, # moderate anemia, $ severe anemia

**Fig. 5:**
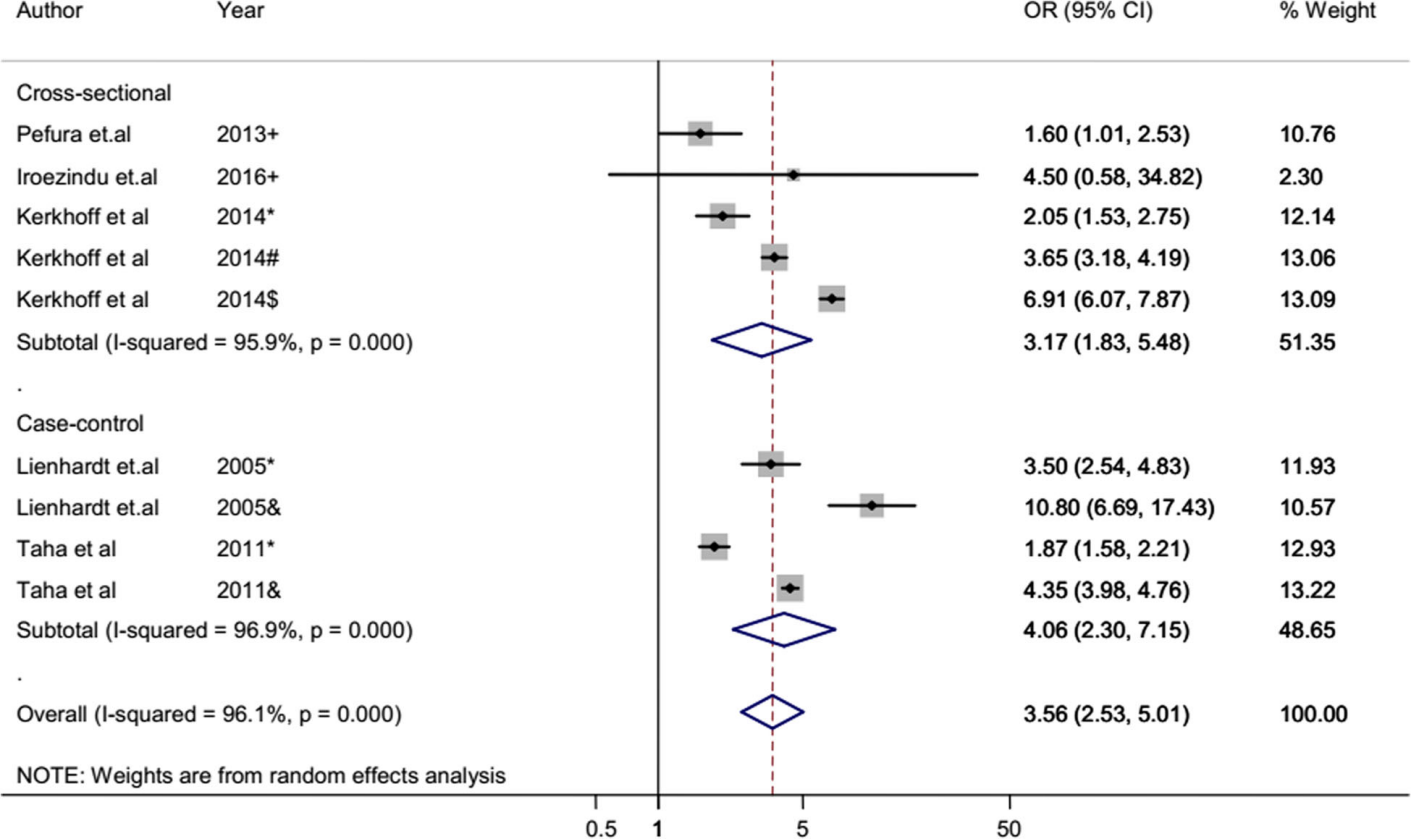
Forest plot of sub-group analysis pooled effect size (OR) estimates of anemia for tuberculosis infection by study design. The scale in the *x*-axis represents OR risk estimate of the studies. The hard line represents the odd ratio value showing no association (odds ratio of 1). The dashed line represents the pooled point estimate of OR of anemia for the tuberculosis infection. The black dot at the center of the gray box represents the point OR estimate of each study, and the line indicates the 95% confidence interval of the estimates. The gray boxes represent the weight of each study contributing to the pooled OR estimate. The first two blue diamonds represent the 95% confidence interval for sub group analysis pooled OR estimate and the last blue diamond represents the 95% confidence interval for overall pooled OR estimate. OR odds ratio, CI confidence interval, *I*^2^ shows the heterogeneity of the included studies, *p* indicates the statistical significance of the heterogeneity, + anemia severity not defined, * mild anemia, & moderate and severe anemia, # moderate anemia, $ severe anemia

**Fig. 6:**
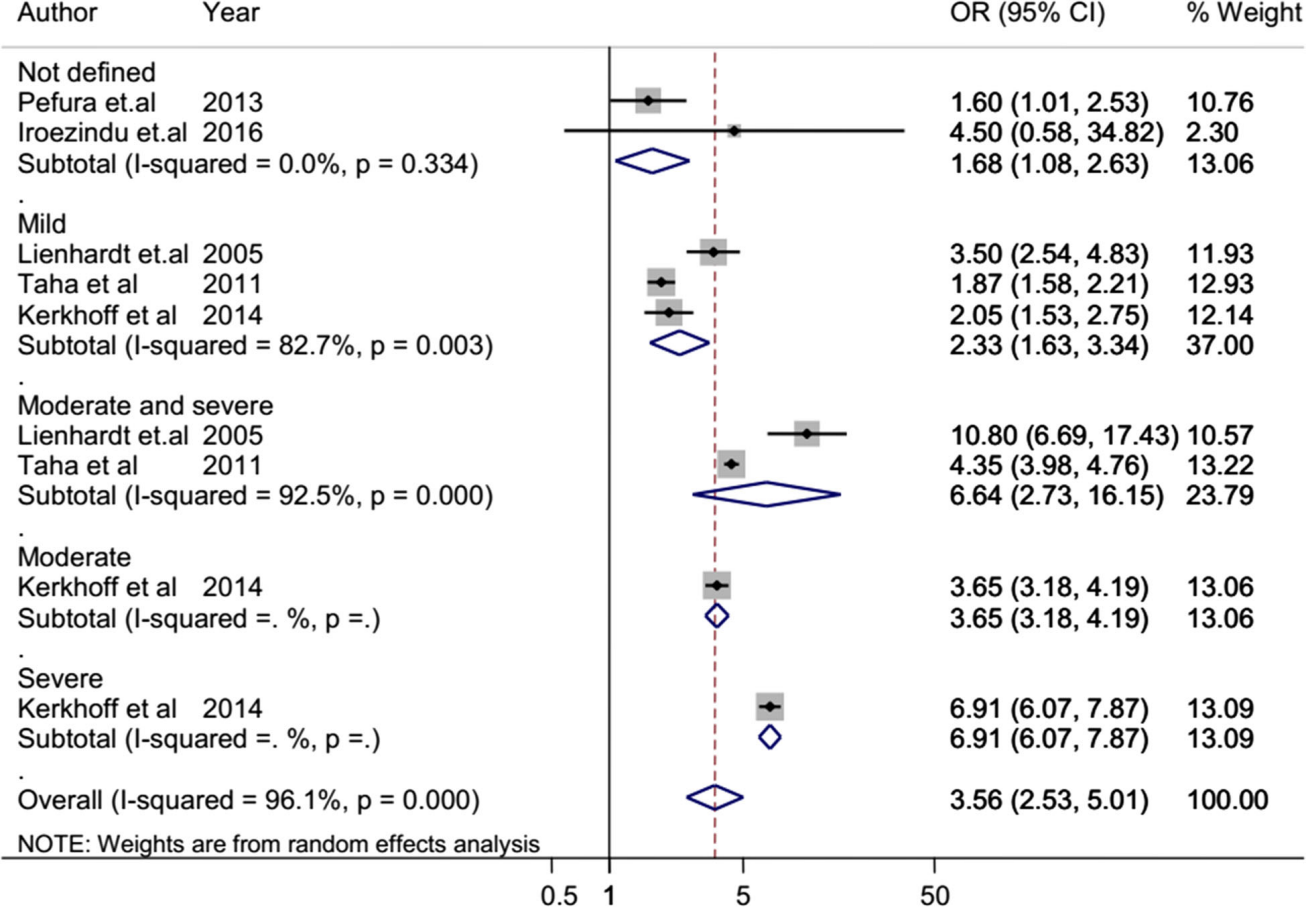
Forest plot of case-control and cross-sectional studies; sub-group analysis pooled effect size (OR) estimates of anemia for TB by anemia severity. The scale in the *x*-axis represents OR risk estimate of the studies. The hard line represents the OR value showing no association (OR of 1). The dashed line represents the pooled point estimate of OR of anemia for the TB. The black dot at the center of the gray box represents the point OR estimate of each study, and the line indicates the 95% confidence interval of the estimates. The gray boxes represent the weight of each study contributing to the pooled OR estimate. The first five blue diamonds represent the 95% confidence interval for subgroup analysis pooled OR estimate, and the last blue diamond represents the 95% confidence interval for overall pooled OR estimate. OR odds ratio, *CI* confidence interval, *I*^2^ shows the heterogeneity of the included studies, *p* indicates the statistical significance of the heterogeneity

**Fig. 7:**
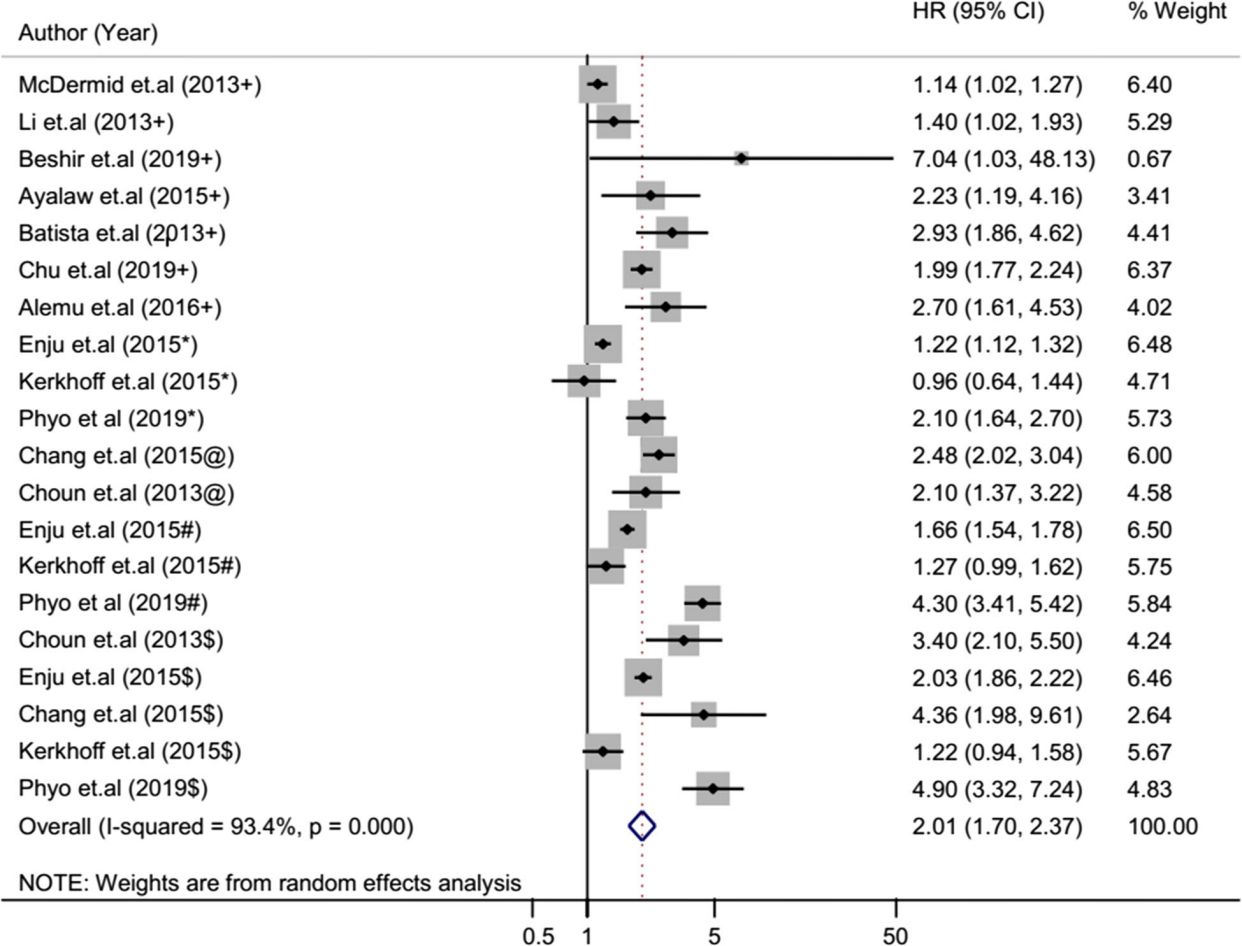
Forest plot of cohort studies; pooled effect size (HR) estimates of anemia for the development of TB. The scale in the *x*-axis represents HR risk estimate of the studies. The hard line represents the HR value showing no association (HR of 1). The dashed line represents the pooled point estimate of HR of anemia for the TB infection. The black dot at the center of the gray box represents the point HR estimate of each study, and the line indicates the 95% confidence interval of the estimates. The gray box represents the weight of each study contributing to the pooled HR estimate. The blue diamond represents the 95% confidence interval of the pooled HR estimate. *HR* hazard ratio, *CI* confidence interval, *I*^2^ shows the heterogeneity of the included studies, *p* indicates the statistical significance of the heterogeneity, + anemia severity not defined, * mild anemia, @ mild and moderate anemia, # moderate anemia, $ severe anemia

**Fig. 8:**
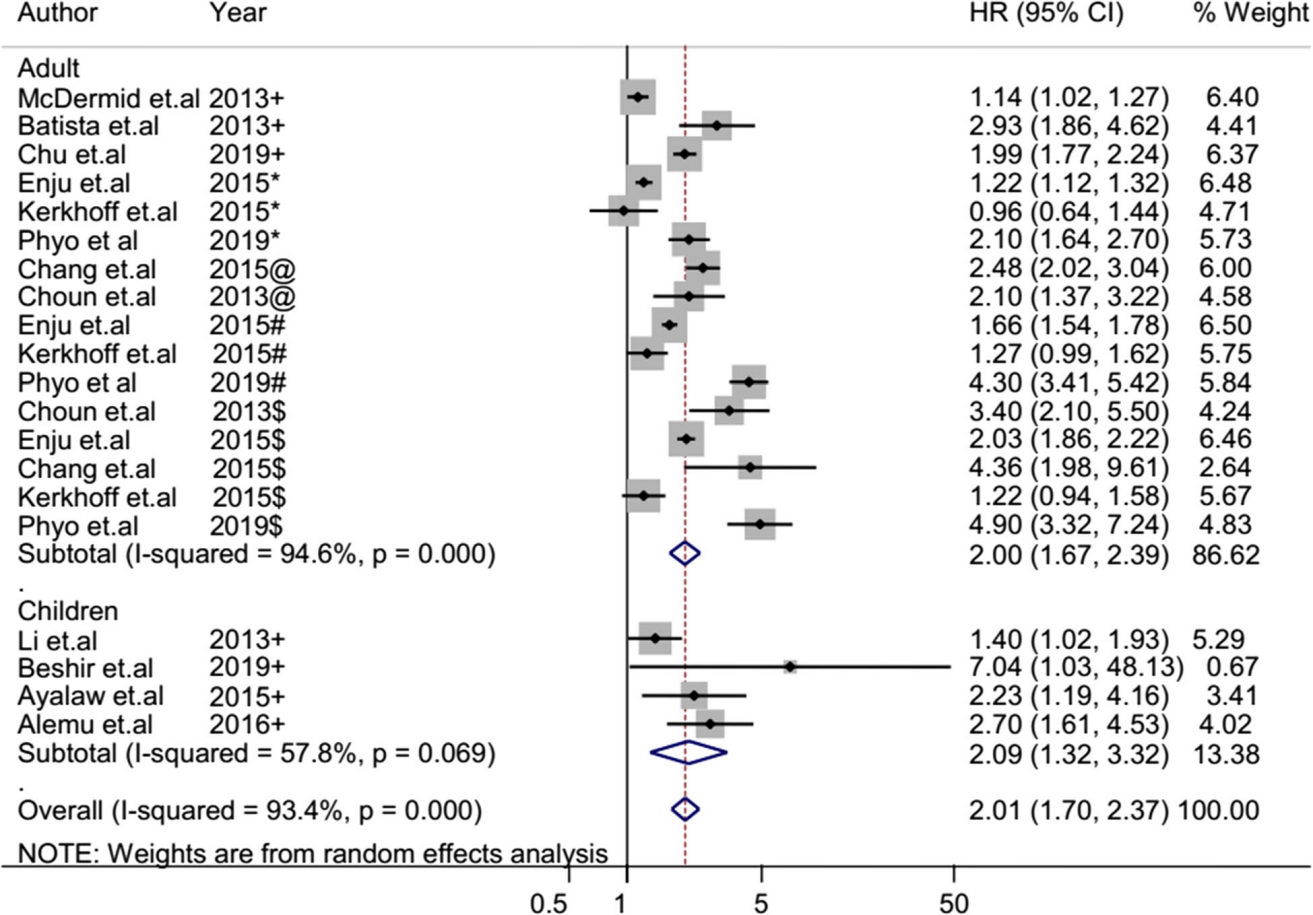
Forest plot of cohort studies; sub-group analysis pooled effect size (HR) estimates of anemia for TB by participant age. The scale in the *x*-axis represents HR risk estimate of the studies. The hard line represents the odd ratio value showing no association (HR of 1). The dashed line represents the pooled point estimate of HR of anemia for the TB infection. The black dot at the center of the gray box represents the point HR estimate of each study, and the line indicates the 95% confidence interval of the estimates. The gray boxes represent the weight of each study contributing to the pooled HR estimate. The first two blue diamonds represent the 95% confidence interval for subgroup analysis pooled HR estimate, and the last blue diamond represents the 95% confidence interval for overall pooled HR estimate. HR hazard ratio, CI confidence interval, *I*^2^ shows the heterogeneity of the included studies, *p* indicates the statistical significance of the heterogeneity, + anemia severity not defined, * mild anemia, @ mild and moderate anemia, # moderate anemia, $ severe anemia

**Fig. 9:**
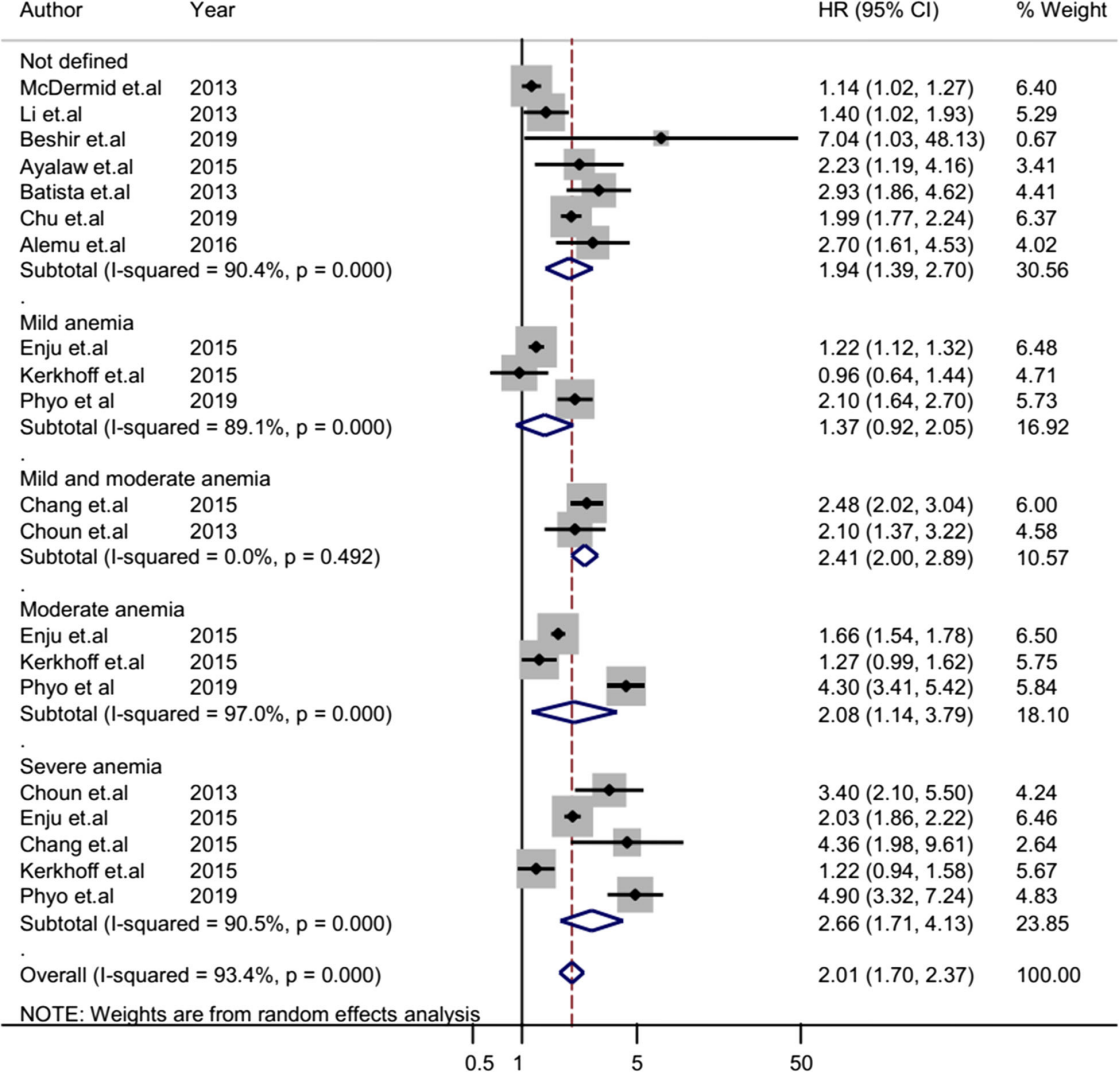
Forest plot of cohort studies; sub-group analysis pooled effect size (HR) estimates of anemia for TB by anemia severity. The scale in the *x*-axis represents HR risk estimate of the studies. The hard line represents the HR value showing no association (HR of 1). The dashed line represents the pooled point estimate of HR of anemia for the TB infection. The black dot at the center of the gray box represents the point HR estimate of each study, and the line indicates the 95% confidence interval of the estimates. The gray boxes represent the weight of each study contributing to the pooled HR estimate. The first five blue diamonds represent the 95% confidence interval for subgroup analysis pooled HR estimate, and the last blue diamond represents the 95% confidence interval for overall pooled HR estimate. *HR* hazard ratio, *CI* confidence interval, I-squared shows the heterogeneity of the included studies, *p* indicates the statistical significance of the heterogeneity

The overall heterogeneity of I-squared statistics showed substantial heterogeneity for studies pooled by both OR and HR (I-squared = 96.1% and 93.4%, respectively) (Figs. 4 and 7). Therefore, the subgroup analysis was performed for pooled studies showing high heterogeneity. Studies pooled by OR were sub-analyzed by their study design and anemia severity status (Figs. 5 and 6). However, there was still a substantial heterogeneity except for sub-group analysis by anemia severity which showed that heterogeneity was not observed in studies in which anemia severity was not defined (Fig. 6). On the other hand, cohort studies pooled by HR were sub-analyzed by their participants’ age and anemia severity status. The result showed that heterogeneity was not observed in studies conducted children and in studies where anemia severity was mild and moderate (Figs. 8 and 9). Sensitivity and heterogeneity test of the included studies was also done to test the effect of each study on the pooled effect size by excluding each study step by step. But the sensitivity results showed that no study was the prime determinants of the pooled effect size (all studies had nearly equal contribution for the pooled estimate). The heterogeneity test also showed that a single study was not the source of heterogeneity (there were no significance reduction of heterogeneity by omitting each study step by step) (Tables 4 and 5).

**Table 4:** Sensitivity and heterogeneity test of pooled case-control and cross-sectional studies

Study omitted	OR (95% CI)	Heterogeneity: *I*^2^%
Lienhardt et al. (2005) mild anemia [27]	3.57 (2.46–5.19)	96.6
Lienhardt et al. (2005) moderate and severe anemia [27]	3.13 (2.20–4.44)	96.3
Taha et al. (2011) mild anemia [28]	3.93 (2.90–5.32)	93.8
Taha et al. (2011) moderate and severe anemia [28]	3.47 (2.19–5.50)	96.5
Kerkhoff et al. (2014) mild anemia [11]	3.85(2.69–5.50)	96.2
Kerkhoff et al. (2014) moderate anemia [11]	3.56 (2.35–5.40)	96.6
Kerkhoff et al. (2014) severe anemia [11]	3.22 (2.30–4.49)	94.3
Pefura et al. (2013) anemia severity not defined [29]	3.92 ( 2.76–5.56)	96.3
Iroezindu et al. (2016) anemia severity not defined [24]	3.54 (2.51–5.01)	96.6
Combined	3.56 (2.53–5.01)	96.1

*OR* odds ratio, *CI* confidence interval, *I*^2^ shows the heterogeneity of the included studies when the corresponding study is omitted

**Table 5:** Sensitivity and heterogeneity test of the included cohort studies pooled by HR

Study omitted		Heterogeneity: *I*^2^%
McDermid et al. (2013)^a^ [32]	2.08 (1.76–2.46)	92.4
Li et al. (2013)^a^[33]	2.05 (1.72–2.43)	93.4
Beshir et al. (2019)^a^ [10]	1.99 (1.68–2.35)	93.7
Ayalaw et al. (2015)^a^[30]	2.00 (1.69–2.37)	93.7
Batista et al. (2013)^a^[31]	1.97 (1.66–2.33)	93.6
Chu et al. (2019) ^a^ [17]	2.01 (1.69–2.41)	93.5
Alemu et al. (2016)^a^ [22]	1.98 (1.67–2.35)	93.7
Enju et al. (2015)^b^ [21]	2.07 (1.75–2.46)	91.9
Kerkhoff et al. (2015)^b^ [20]	2.08 (1.76–2.46)	93.6
Phyo et al. (2019)^b^ [23]	2.00 (1.68–2.38)	93.7
Chang et al. (2015)^c^ [34]	1.98 (1.67–2.35)	93.4
Choun et al. (2013) ^c^ [35]	2.00 (1.69–2.37)	93.7
Enju et al. (2015) ^d^ [21]	2.05 (1.69–2.50)	93.7
Kerkhoff et al. (2015) ^d^ [20]	2.06 (1.74–2.45)	93.6
Phyo et al. (2019) ^d^ [23]	1.89 (1.62–2.21)	91.8
Choun et al. (2013) ^e^ [35]	1.96 (1.66–2.32)	93.5
Enju et al. (2015) ^e^ [21]	2.01 (1.68–2.41)	93.1
Chang et al. (2015) ^e^ [34]	1.96 (1.66–2.32)	93.6
Kerkhoff et al. (2015) ^e^ [20]	2.07 (1.74–2.45)	93.6
Phyo et al. (2019) ^e^ [23]	1.91 (1.62–2.25)	93.0
Combined	2.01 (1.70–2.37)	93.4

*HR* hazard ratio, *CI* confidence interval, *I*^2^ shows the heterogeneity of the included studies when the corresponding study is omitted

^a^Anemia severity not defined

^b^Mild anemia

^c^Mild and moderate anemia

^d^Moderate anemia

^e^Severe anemia

### Pooled risk estimate of anemia for TB: cross-sectional and case-control studies

According to the pooled OR of cross-sectional and case-control studies, the odds of TB infection among anemic patients is 3.56 times higher than non-anemic patients (95% CI 2.5–5.0). The sub-group analysis of these studies by anemia severity status also revealed that the odds of contracting TB was increased in severely anemic patients. The odd of TB among mild anemic patients is 2.33 times higher than non-anemic patients, while in moderately and severely anemic patients, it was 3.65 and 3.91 times higher than non-anemic patients, respectively (Fig. 6).

### Pooled risk estimate of anemia for TB based on cohort studies

Ten cohort studies assessed the risk of anemia for TB by using HR effected size and analyzed these studies together to get the pooled effect size of anemia on TB by using HR. Accordingly, their pooled HR showed that the hazared of TB among anemic patients was 2.01 times higher than non-anemic patients (95% CI 1.70–2.37) (Fig. 7). The sub-analysis of the cohort studies by age group revealed that there was no significant change of risk of anemia for TB between anemic children and anemic adults (Fig. 8). However, the sub-analysis of these cohort studies showed that the hazared of TB was increased with anemia severity (Fig. 9). The hazard of TB among mild anemic patients was not statistically significance compared to non-anemic patients (HR 1.4, 95% CI 0.9–1.9). However, the hazard of TB among moderate anemic patients was 2.08 (95% CI 1.14–3.79) times higher than non-anemic patients, whereas in severe anemic patients, it was 2.66 (95% CI 1.71–4.13) times higher compared to non-anemic patients.

## Discussion

To the best of our information, this is the first systematic review and meta-analysis conducted to determine the pooled risk factor of anemia for TB. Anemia defined by low Hb or red blood cell (RBC) concentration is a major hematological finding in chronic diseases [39, 40]. It is also a known risk factor for some chronic diseases [41]. In the current systematic review and meta-analysis, majority of the included studies showed that anemia was a higher predicator of TB [10, 11, 17, 22, 27–29, 30, 31]. However, some studies showed that anemia was not the risk of TB [20, 24, 33]. The reason for discrepancy might be different in sample size, study design, diagnostic method, and geographical location.

But, according to the current systematic review and meta-analysis pooled effect size estimate, anemia was the risk factor of TB. The pooled effect of cross-sectional and case-control studies showed that the odds of TB among anemic patients were 3.56 times higher than non-anemic patients. Indeed, this may not show the cause and effect association due to the limitation of study design (is anemia the cause of TB infection? or is TB disease the cause of anemia?).

However, pooled analysis of cohort studies, which show the cause and effect association, also revealed that anemia was a risk for TB. According to the pooled effect estimate of 10 cohort studies analyzed by HR, the hazard of TB among anemic patients was 2.01 times higher than non-anemic patients. This might be due to that anemic patients might be nutritionally imbalanced and immuno-compromised. Anemia was used as indirect assessment of nutritional and immune status of the individuals. Most of the included studies did not report the type of anemia. However, the WHO reported that 50% of the cases of anemia are due to iron deficiency [42] which might be true for the included studies. Successful transmission of TB is influenced by a variety of conditions, including proximity and duration of contact with an individual with active TB disease, and the immune-competency of the individual infected with tuberculosis [43]. Individuals with a weak immune response (immune compromise individuals) are at risk of TB [44].

Iron was confirmed to be a vital element not only for erythropoiesis, but also for immune system development and play an important role in the integrity of the immune system; and its deficiency can cause impairment of immunity. Ekiz et al. suggests that the important immunogenic mechanisms like humoral, cell-mediated, and nonspecific immunity and the activity of cytokines are influenced by iron deficiency anemia. Especially, the percentage of monocytes with oxidative burst activity and the ratio of monocytes with phagocytic activity were highly reduced in iron deficiency anemia [13]. Macrophage phagocytic activities are important immunological response in controlling of TB infection by forming granulomas which is an aggregate of immune cells and walls of the mycobacterium which limiting further replication and spread of the tubercle bacilli [44, 45]. Aly et al. and Das et al. also showed that iron deficiency anemia impaired cell-mediated immune response specifically T cell-mediated immunity [46, 47]. A review by Stephen stated that even all studies did not show a consistence result, there were an impairment of polymorph neutrophil function and intracellular bacteriocidal activity of immunological cells in iron deficient individuals [1]. Iron status may also modulate the type of immune response mounted through its influence on the body’s cytokine profile. Experimental evidence has shown that iron deficiency changes the balance between Th1 and Th2 cytokines, promoting a dominant Th2 response that has been associated with clinical TB disease [48]. Generally, morbidity from infectious disease is increased in iron-deficient populations, because of the adverse effect of iron deficiency on the immune system [49, 50]. Therefore, the anemic patients probably might have impaired or modulated immune system that favors the replication of TB.

The other cause of anemia are the presence of other micronutrient deficiencies, including vitamins A and B12, folate, riboflavin, and copper [42]. These micronutrient deficiencies can also cause immunological impairment [51]. According to Erkurt et al, vitamin B12 has important immunomodulatory effects on cellular immunity, and abnormalities in the immune system in pernicious anemia are restored by vitamin B12 replacement therapy [12].

The other possible explanation might be the direct involvement of RBCs in maintaining of the innate and adaptive immune system. Evidence shows that RBCs are modulators of T cell proliferation. In particular, RBCs are able to enhance T cell expansion and survival by inhibiting activation-induced T cell death, an effect possibly associated with a decrease of oxidative stress within activated T cells. Optimal T cell proliferation and survival were only observed with intact RBCs and when RBCs were in close contact or proximity with activated T cells [52].

In the current systematic and meta-analysis, the burden of TB was increased with anemia severity. According to the pooled effect estimate of case-control and cross-sectional studies, the odds of TB among mild anemic patients is 2.33 times higher than non-anemic patients, while in moderately and severely anemic patients, it was 3.65 and 6.91 times higher than non-anemic patients, respectively. The included cohort studies also revealed that the hazard of TB was increased from 1.37 times (statistically insignificance) to 2.08 times and 2.66 times among mild, moderately, and severely anemic patients compared to non-anemic patients, respectively. One in vitro experimental study conducted by Bishlawy and IM EL showed that Hb is strongly bacteriostatic. According to this experiment, a drop of washed RBCs is put in a Petri dish containing nutrient agar inoculated with staphylococci and incubated for 24–48 h at 37 °C. The RBCs used were undiluted or 50% diluted with saline. The result showed undiluted washed RBCs blocked bacterial growth, but impaired by dilution [53]. This justifies the finding, the higher incidence of TB among anemic patients than non-anemic patients, and why it was increased in moderate and severe anemic patients compared to mild anemic patients. In anemic patients, there are low RBCs (low concentration of Hb), especially in moderate and severe anemic patients. Therefore, anemic patients might have disturbed immune system and low bacteriocidal activity due to low Hb concentration, which enhances the growth of TB.

Generally, anemia is a risk factor for TB; this is because anemic patients might be immuno-suppressed and susceptible to TB, and it is known that TB is common in immuno-compromised population patients [54].

### Strength and limitation of the study

One of the strengths of this review was being the first systematic review and meta-analysis to determine the pooled risk estimate of anemia for TB. Moreover, the review was conducted according to the preferred reporting items for systematic review and meta-analysis (PRISMA-P statement) protocol. However, this review had limitations. The extent of heterogeneity between included studies was high. We were unable to get adjusted risk ratio from some of the included studies. Therefore, we used crude risk measures from these articles. The other limitation of this systematic review and meta-analysis was, only articles published in English were used for our literature search. Most of the included studies were conducted in Africa which might cause geographical bias.

## Conclusion

Anemia is a major public health concerns that may predispose an individual to some infectious diseases including TB. According to the current systematic and meta-analysis, anemia was the risk factor of TB and the risk was increased with anemia severity. Diagnosis of anemia with Hb measurement is a low cost and more widely available in clinical settings to know the anemic status of the individual. Anemia screening, early diagnose and treatment may reduce the magnitude TB at the community level. Therefore, health professionals should treat anemia as early as possible and policy makers should consider anemic patients as risk for TB and established screening guideline.

## Data Availability

The datasets used and/or analyzed during the current study are available from the corresponding author and can access on reasonable request.

## Abbreviations

CI: Confidence interval
Hb: Hemoglobin
HIV: Human immunodeficiency virus
HR: Hazard ratio
JBI: Joanna Brigg Institute
MeSH: Medical Subject Headings
OR: Odds ratio
PRISMA: Preferred Reporting Items for Systematic Reviews and Meta-Analyses
RBCs: Red blood cells
RR: Risk ratio
TB: Tuberculosis
WHO: World Health Organization

## References

[1] Stevens GA, Finucane MM, De-Regil LM, Paciorek CJ, Flaxman SR, Branca F (2013). Global, regional, and national trends in haemoglobin concentration and prevalence of total and severe anaemia in children and pregnant and non-pregnant women for 1995–2011: a systematic analysis of population-representative data. Lancet Glob Health.

[2] WHO, Haemoglobin concentrations for the diagnosis of anaemia and assessment of severity; World Health Organization; Available from: https://www.who.int/vmnis/indicators/haemoglobin.pdf; Access date; October 20, 2020.

[3] De Benoist B, Cogswell M, Egli I, McLean E (2008). Worldwide prevalence of anaemia 1993-2005.

[4] Cote C, Zilberberg MD, Mody SH, Dordelly LJ, Celli B (2007). Haemoglobin level and its clinical impact in a cohort of patients with COPD. Eur Respir J.

[5] Barzegari S, Afshari M, Movahednia M, Moosazadeh M (2019). Prevalence of anemia among patients with tuberculosis: a systematic review and meta-analysis. Indian J Tuberc.

[6] Nagu TJ, Spiegelman D, Hertzmark E, Aboud S, Makani J, Matee MI, et al. Anemia at the initiation of tuberculosis therapy is associated with delayed sputum conversion among pulmonary tuberculosis patients in Dar-es-Salaam, Tanzania. PloS one. 2014;9(3). 10.1371/journal.pone.0091229 PMC3958362 24642636

[7] Hella J, Cercamondi CI, Mhimbira F, Sasamalo M, Stoffel N, Zwahlen M (2018). Anemia in tuberculosis cases and household controls from Tanzania: contribution of disease, coinfections, and the role of hepcidin. PLoS One.

[8] Bashir BA, Abdallah SA, Mohamedani AA (2015). Anemia among patients with pulmonary tuberculosis in port Sudan, eastern Sudan. Int J Recent Sci Res.

[9] Oliveira MG, Delogo KN (2014). Oliveira HMdMG, Ruffino-Netto A, Kritski AL, Oliveira MM. Anemia in hospitalized patients with pulmonary tuberculosis. J Bras Pneumol.

[10] Beshir MT, Beyene AH, Tlaye KG, Demelew TM (2019). Incidence and predictors of tuberculosis among HIV-positive children at Adama Referral Hospital and Medical College, Oromia, Ethiopia: a retrospective follow-up study. Epidemiol Health.

[11] Kerkhoff AD, Wood R, Vogt M, Lawn SD. Predictive value of anemia for tuberculosis in HIV-infected patients in Sub-Saharan Africa: an indication for routine microbiological investigation using new rapid assays. Journal of acquired immune deficiency syndromes (1999). 2014;66(1):33-40. 10.1097/QAI.0000000000000091 PMC3981888 24346639

[12] Erkurt MA, Aydogdu I, Dikilitaş M, Kuku I, Kaya E, Bayraktar N (2008). Effects of cyanocobalamin on immunity in patients with pernicious anemia. Med Princ Pract.

[13] Ekiz C, Agaoglu L, Karakas Z, Gurel N, Yalcin I (2005). The effect of iron deficiency anemia on the function of the immune system. Hematol J.

[14] Nagu TJ, Spiegelman D, Hertzmark E, Aboud S, Makani J, Matee MI (2014). Anemia at the initiation of tuberculosis therapy is associated with delayed sputum conversion among pulmonary tuberculosis patients in Dar-es-Salaam. Tanzania PloS one.

[15] Devi U, Rao CM, Srivastava VK, Rath PK, Das BS (2003). Effect of iron supplementation on mild to moderate anaemia in pulmonary tuberculosis. Br J Nutr.

[16] Umakanth M (2017). Anemia among tuberculosis patient in teaching hospital Batticaloa, Sri Lanka. Int J Med Heal Res.

[17] Chu K-A, Hsu C-H, Lin M-C, Chu Y-H, Hung Y-M, Wei JC-C (2019). Association of iron deficiency anemia with tuberculosis in Taiwan: a nationwide population-based study. PLoS One.

[18] WHO, Systematic screening for active tuberculosis: principles and recommendations, World Health Organization, 2013, Available from https://www.who.int/tb/publications/Final_TB_Screening_guidelines.pdf, Access date November 30, 2019. 25996015

[19] Gurvits GE, Lan G (2014). Enterolithiasis. World J Gastroenterol.

[20] Kerkhoff AD, Wood R, Cobelens FG, Gupta-Wright A, Bekker LG, Lawn SD (2015). The predictive value of current haemoglobin levels for incident tuberculosis and/or mortality during long-term antiretroviral therapy in South Africa: a cohort study. BMC Med.

[21] Enju L, MAKUBI A, DRAIN P, SPIEGELMAN D, SANDO D, Nan L, et al. Tuberculosis incidence rate and risk factors among HIV-infected adults with access to antiretroviral therapy in Tanzania. AIDS (London, England). 2015;29(11):1391-9. 10.1097/QAD.0000000000000705 PMC4576970 26091295

[22] Alemu YM, Andargie G, Gebeye E (2016). High incidence of tuberculosis in the absence of isoniazid and cotrimoxazole preventive therapy in children living with HIV in Northern Ethiopia: a retrospective follow-up study. PLoS One.

[23] Phyo K, Oo M, Harries A, Saw S, Aung T, Moe J (2019). High prevalence and incidence of tuberculosis in people living with the HIV in Mandalay, Myanmar, 2011–2017. Int J Tuberc Lung Dis.

[24] Iroezindu M, Ofondu E, Mbata G, Van Wyk B, Hausler H, Au D (2016). Factors associated with prevalent tuberculosis among patients receiving highly active antiretroviral therapy in a Nigerian tertiary hospital. Ann Med Health Sci Res.

[25] Moher D, Shamseer L, Clarke M, Ghersi D, Liberati A, Petticrew M (2015). Preferred reporting items for systematic review and meta-analysis protocols (PRISMA-P) 2015 statement. Syst Rev.

[26] Moola S, Munn Z, Tufanaru C, Aromataris E, Sears K, Sfetcu R, Currie M, Qureshi R, Mattis P, Lisy K, Mu P-F. Chapter 7: systematic reviews of etiology and risk. In: Aromataris E, Munn Z (Editors). Joanna Briggs Institute Reviewer’s Manual. The Joanna Briggs Institute, 2017. Available from https://reviewersmanual.joannabriggs.org/: Access date December 25, 2019.

[27] Lienhardt C, Fielding K, Sillah J, Bah B, Gustafson P, Warndorff D (2005). Investigation of the risk factors for tuberculosis: a case–control study in three countries in West Africa. Int J Epidemiol.

[28] Taha M, Deribew A, Tessema F, Assegid S, Duchateau L, Colebunders R (2011). Risk factors of active tuberculosis in people living with HIV. Ethiop J Health Sci.

[29] Pefura Yone EW, Kengne AP, Moifo B, Kuaban C (2013). Prevalence and determinants of extrapulmonary involvement in patients with pulmonary tuberculosis in a Sub-Saharan African country: a cross-sectional study. Scand J Infect Dis.

[30] Ayalaw SG, Alene KA, Adane AA (2015). Incidence and predictors of tuberculosis among HIV positive children at University of Gondar Referral Hospital, northwest Ethiopia: a retrospective follow-up study. Int Scholarly Res Notices.

[31] Batista JdAL, Maruza M, de Alencar Ximenes RA, Santos ML, Montarroyos UR, de Barros Miranda-Filho D, et al. Incidence and risk factors for tuberculosis in people living with HIV: cohort from HIV referral health centers in Recife, Brazil. PloS one. 2013;8(5):e63916. 10.1371/journal.pone.0063916 PMC3651200 23675515

[32] McDermid JM, Hennig BJ, van der Sande M, Hill AV, Whittle HC, Jaye A (2013). Host iron redistribution as a risk factor for incident tuberculosis in HIV infection: an 11-year retrospective cohort study. BMC Infect Dis.

[33] Li N, Manji KP, Spiegelman D, Muya A, Mwiru RS, Liu E, Chalamilla G, Fawzi WW, Duggan C. Incident tuberculosis and risk factors among HIV-infected children in Tanzania. AIDS. 2013;27(8):1273–81. 10.1097/QAD.0b013e32835ecb24 PMC4742782 23343909

[34] Chang CA, Meloni ST, Eisen G, Chaplin B, Akande P, Okonkwo P (2015). Tuberculosis incidence and risk factors among human immunodeficiency virus (HIV)-infected adults receiving antiretroviral therapy in a large HIV program in Nigeria. Open Forum Infect Dis.

[35] Choun K, Thai S, Pe R, Lorent N, Lynen L, van Griensven J (2013). Incidence and risk factors for tuberculosis in HIV-infected patients while on antiretroviral treatment in Cambodia. Trans R Soc Trop Med Hyg.

[36] Klote MM, Agodoa LY, Abbott KC (2006). Risk factors for Mycobacterium tuberculosis in US chronic dialysis patients. Nephrol Dialysis Transplantation.

[37] Karstaedt A, Bolhaar M (2014). Tuberculosis in older adults in Soweto, South Africa. Int J Tuberc Lung Dis.

[38] Shu C-C, Wu V-C, Yang F-J, Pan S-C, Lai T-S, Wang J-Y (2012). Predictors and prevalence of latent tuberculosis infection in patients receiving long-term hemodialysis and peritoneal dialysis. PLoS One.

[39] Gonzalez-Casas R, Jones EA, Moreno-Otero R (2009). Spectrum of anemia associated with chronic liver disease. World J Gastroenterol: WJG.

[40] Weiss G, Goodnough LT (2005). Anemia of chronic disease. N Engl J Med.

[41] Sarnak MJ, Tighiouart H, Manjunath G, MacLeod B, Griffith J, Salem D (2002). Anemia as a risk factor for cardiovascular disease in the atherosclerosis risk in communities (aric) study. J Am Coll Cardiol.

[42] WHO. Worldwide prevalence of anaemia 1993–2005: WHO global database on anaemia. World Health Organization, 2015. Available from: http://apps.who.int/iris/bitstream/handle/10665/43894/9789241596657_eng.pdf?ua=1 : Access Date: November 1, 2018.

[43] Sia JK, Rengarajan J (2019). Immunology of Mycobacterium tuberculosis infections. Gram-Positive Pathogens.

[44] Ahmad S (2011). Pathogenesis, immunology, and diagnosis of latent Mycobacterium tuberculosis infection. Clin Dev Immunol.

[45] Flynn JL, Chan J (2001). Immunology of tuberculosis. Annu Rev Immunol.

[46] Aly SS, Fayed HM, Ismail AM, Hakeem GLA (2018). Assessment of peripheral blood lymphocyte subsets in children with iron deficiency anemia. BMC Pediatr.

[47] Das I, Saha K, Mukhopadhyay D, Roy S, Raychaudhuri G, Chatterjee M (2014). Impact of iron deficiency anemia on cell-mediated and humoral immunity in children: A case control study. J Natural Sci Biol Med.

[48] Cronjé L (2005). N E, EK, L. B. Iron and iron chelating agents modulate Mycobacterium tuberculosis growth and monocyte-macrophage viability and effector functions. FEMS Immunol Med Microbiol.

[49] Cherayil BJ (2010). Iron and immunity: immunological consequences of iron deficiency and overload. Arch Immunol Ther Exp (Warsz).

[50] World Health Organization. Iron Deficiency Anaemia Assessment, Prevention and Control A guide for programme managers. World Health Organization; 2001. Availabel from: https://www.who.int/nutrition/publications/en/ida_assessment_prevention_control.pdf: Access date: October, 19, 2020.

[51] Saeed F, Nadeem M, Ahmed RS, Tahir Nadeem M, Arshad MS, Ullah A (2016). Studying the impact of nutritional immunology underlying the modulation of immune responses by nutritional compounds–a review. Food Agricultural Immunol.

[52] Buttari B, Profumo E, Riganò R (2015). Crosstalk between red blood cells and the immune system and its impact on atherosclerosis. Biomed Res Int.

[53] Bishlawy IE (1999). Red blood cells, hemoglobin and the immune system. Med Hypotheses.

[54] Ibrahim B, Sani AM, Timothy B (2016). Prevalence of glucose-6-phosphate dehydrogenase deficiency among children aged 0-5 years infected with Plasmodium falciparum in Katsina State, Nigeria. Adv Biochem.

